# Scalable live-attenuated SARS-CoV-2 vaccine candidate demonstrates preclinical safety and efficacy

**DOI:** 10.1073/pnas.2102775118

**Published:** 2021-06-30

**Authors:** Ying Wang, Chen Yang, Yutong Song, J. Robert Coleman, Marcin Stawowczyk, Juliana Tafrova, Sybil Tasker, David Boltz, Robert Baker, Liliana Garcia, Olivia Seale, Anna Kushnir, Eckard Wimmer, Steffen Mueller

**Affiliations:** ^a^Codagenix Inc., Farmingdale, NY 11735;; ^b^Division of Microbiology and Molecular Biology, Illinois Institute of Technology (IIT) Research Institute, Chicago, IL 60616;; ^c^Department of Microbiology and Immunology, Stony Brook University, Stony Brook, NY 11794

**Keywords:** vaccine, COVID-19, live attenuated, codon deoptimization

## Abstract

Successfully combating the COVID-19 pandemic depends on mass vaccination with suitable vaccines to achieve herd immunity. Here, we describe COVI-VAC, the only live attenuated severe acute respiratory syndrome coronavirus 2 (SARS-CoV-2) vaccine currently in clinical development. COVI-VAC was developed by recoding a segment of the viral spike protein with synonymous suboptimal codon pairs (codon-pair deoptimization), thereby introducing 283 silent (point) mutations. In addition, the furin cleavage site within the spike protein was deleted from the viral genome for added safety of the vaccine strain. Except for the furin cleavage site deletion, the COVI-VAC and parental SARS-CoV-2 amino acid sequences are identical, ensuring that all viral proteins can engage with the host immune system of vaccine recipients. COVI-VAC was temperature sensitive in vitro yet grew robustly (>10^7^ plaque forming units/mL) at the permissive temperature. Tissue viral loads were consistently lower, lung pathology milder, and weight loss reduced in Syrian golden hamsters (*Mesocricetus auratus*) vaccinated intranasally with COVI-VAC compared to those inoculated with wild-type (WT) virus. COVI-VAC inoculation generated spike IgG antibody levels and plaque reduction neutralization titers similar to those in hamsters inoculated with WT virus. Upon challenge with WT virus, COVI-VAC vaccination reduced lung challenge viral titers, resulted in undetectable virus in the brain, and protected hamsters from almost all SARS-CoV-2–associated weight loss. Highly attenuated COVI-VAC is protective at a single intranasal dose in a relevant in vivo model. This, coupled with its large-scale manufacturing potential, supports its potential use in mass vaccination programs.

## Significance

This work demonstrates the feasibility of rationally designing and synthesizing vaccine candidates for testing in response to an emerging disease in real world conditions. Furthermore, using a live attenuated codon-pair–deoptimized virus approach ensures that all components of the host immune system will be engaged, and potential effects from the vector sequences from hybrid live viruses are avoided. Evidence from other codon-deoptimized viruses suggests that COVI-VAC will be resistant to reversion and loss of potency due to antigenic drift. The ease of large-scale virus growth under permissive conditions coupled with the potential for single-dose intranasal administration make COVI-VAC an appealing candidate for clinical testing for possible use in mass immunization programs.

In just the first 9 mo of the severe acute respiratory syndrome coronavirus 2 (SARS-CoV-2) pandemic, the virus killed more people than the 2009 influenza epidemic in its entirety, and within 1 y, more than 74 million people have been infected worldwide while the number continues to rise (1). With 7 billion people in the world, multiple vaccines are needed to fight SARS-CoV-2 that are safe and effective for people of any age, demographic group, or medical condition. Furthermore, mass vaccinations require easy and inexpensive delivery that can be administered noninvasively (e.g., intranasally [IN] or orally), if possible.

With a deeper understanding of the immune correlates of protection from SARS-CoV-2 infection still emerging, a vaccine that presents more than one SARS-CoV-2 protein or antigen to the host is appealing because it circumvents the need to define a specific target. Live attenuated vaccines (LAVs) are particularly attractive, as they activate all branches of the host immune system (humoral, innate, and cellular) (2, 3). Also, a vaccine that can present all SARS-CoV-2 antigens to the host is optimal because it can both induce a broad immune response and is less likely to lose significant potency due to the antigenic drift that we are already witnessing (4–6).

We have conducted preclinical testing of a SARS-CoV-2 LAV, designed by the “synthetic attenuated virus engineering” (SAVE) strategy, which we propose has led to a live COVID-19 vaccine candidate (COVI-VAC). SAVE takes advantage of naturally occurring codon pair bias in human cells and “deoptimized” viral sequences (7, 8). Specifically, codon pair bias describes an inflexible rule in all tested organisms that the juxtaposition of two codons in an open reading frame (ORF) can lead to favorable and unfavorable codon pairs (7). The biological difference between favorable and unfavorable codon pairs is small. However, if by computer design and genome synthesis the number of unfavorable codon pairs in an ORF is increased (“codon-pair deoptimization”), expression of the ORF is decreased. Remarkably, an excess of synonymous nonfavorable codon pairs leads to a nonviable virus (7).

Because viruses use the host cell to translate their genome, genes of wild-type (WT) viruses that infect humans are adapted to the human cell and are efficiently translated. SAVE replaces efficient WT codon pairs in the reading frame of a viral genome with synonymous suboptimal codon pairs leading to reduced protein production in human cells due to slowed translation and/or other mechanisms (9). In codon-pair deoptimization, synonymous codon pairs are relocated. That is, individual codons are not changed, they are simply switched with a synonymous codon at a different location in the RNA to create deoptimized pairing. Hence, the amino acid sequence and codon usage is maintained. By doing so, an LAV genome can be generated by recoding the genome in silico followed by its synthesis in the laboratory. Importantly, the amino acid sequence of viral proteins synthesized by the recoded ORF remains unchanged, and the codon usage of the ORF is also maintained.

In our COVI-VAC, the coding region for the spike protein was recoded. Notably, except for the deleted furin cleavage site, the codon-pair–deoptimized COVI-VAC genome encodes the exact same proteins (same amino acid sequences), and uses the same codons, as the original (WT) virus. Yet, the recoding in COVI-VAC leads to 283 point mutations in the viral genomic sequence, and the virus variant is highly attenuated. Because the amino acid sequence is a perfect match to that of the circulating WT strain, every viral antigen is present, providing the potential for a broad immune response and making the vaccine more likely to retain efficacy if there is genetic drift (3).

Because SAVE vaccine attenuation results from hundreds or thousands of mutations, selective pressure on a particular mutation to revert to WT appears to be reduced (10–14). Deep sequencing of two lineages of a SAVE respiratory syncytial virus (RSV) LAV candidate containing 2,692 point mutations revealed only low-level sporadic mutations after 18 passages at the permissive temperature (14). A second RSV strain containing 1,378 point mutations, was genetically stable for at least eight passages at the permissive temperature with only sporadic, low-level mutations found in P6 deep sequencing and P8 Sanger sequencing (14, 15). Thus, the myriad of mutations that contribute to the attenuated phenotype of SAVE-LAVs also appear to confer high stability with respect to reversion to pathogenicity (10–14).

*SAVE* technology can be applied to any virus that is susceptible to reverse genetics techniques (16–19). First developed in the poliovirus system (7), *SAVE* has been extended to influenza virus, RSV, dengue virus, and Zika virus, among others (16, 17). It can be employed to design and synthesize multiple vaccine candidates from the viral genome sequence alone, or as described here, from the intact virus using a reverse transcription and recovery process. We describe here the *SAVE* design, assembly, and preclinical assessment of a SARS-CoV-2 LAV candidate, COVI-VAC.

## Results

**Fig. 1.**
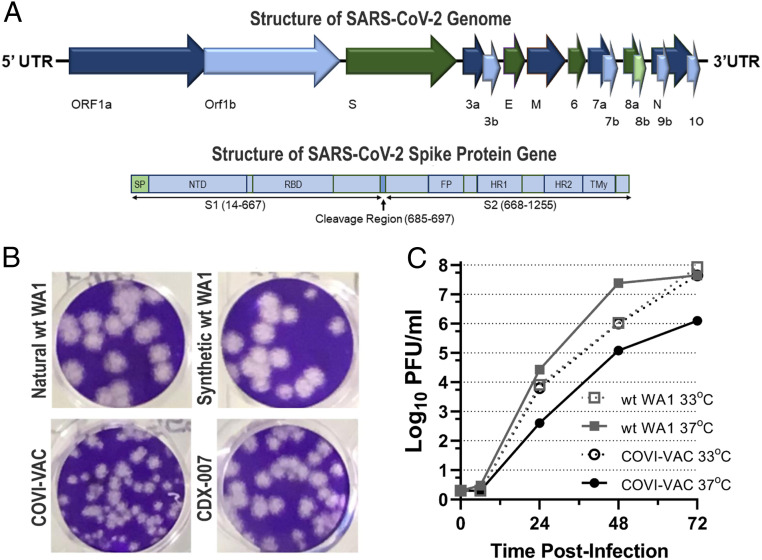
Growth of deoptimized SARS-CoV-2 vaccine candidates. (*A*) Schematic presentation of the SARS-COV-2 viral RNA genome (29,674 nucleotides) and the viral polyprotein, indicating regions encoding numerous viral proteins. Also shown in detail is the coding segment S, the portion of the polyprotein that encodes the spike protein. SP = signal protein; NTD = N-terminal domain RBD = receptor binding domain; FP = fusion peptide; HR1 = heptad repeat 1; HR2 = heptad repeat 2; and TM = transmembrane domain. Indicated in spike *Inset* is the cleavage region that includes the S1/S2 cleavage site that contains the furin cleavage site that was deleted in COVI-VAC and the s2′ cleavage site. (*B*) Plaque phenotypes of natural and synthetic WT SARS-CoV-2 and deoptimized SARS-CoV-2 viruses COVI-VAC and CDX-007 after 67-h incubation in Vero E6 cells. (*C*) Vero cells were infected with WT WA1 or COVI-VAC at 33 or 37 °C. Infected wells were harvested at the indicated time points, and titers were determined by plaque forming assays and reported as log of PFU/mL culture medium.

We applied *SAVE* technology to recover and amplify WT coronavirus strain 2019-nCoV/USA-WA1/2020 (WA1) (distributed by BEI Resources, GenBank accession No. MN985325) and rationally designed two deoptimized LAV candidates against the strain (16). The WT WA1 virus was deposited by the US Centers for Disease Control and Prevention (CDC) and was obtained from BEI Resources. Its sequence was verified by us (20). A schematic of the SARS-CoV-2 genome is shown in Fig. 1*A*. To construct two LAV candidates, a portion of the genome-encoding spike gene sequences (Fig. 1*A*) (21) was replaced by the corresponding codon-pair–deoptimized spike gene sequences. COVI-VAC (strain CDX-005) contains 283, whereas strain CDX-007 contains 149 silent mutations relative to WT WA1 virus. The full-length WT WA1 and deoptimized complementary DNAs were transcribed in vitro to make full-length viral RNA that was electroporated into Vero E6 cells. Transfected cells were incubated for 6 d or until a cytopathic effect (CPE) appeared. Infection medium was collected on days 2, 4, and 6. Virus titer was determined by plaque assay on Vero E6 cells. Plaques were visible as early as day 2 post-transfection, with peak virus generation between days 4 and 6. Though plaques are smaller than those of WT, demonstrating in vitro attenuation, both COVI-VAC and CDX-007 grow robustly in Vero E6 cells, indicating their suitability for scale-up manufacturing (Fig. 1*B*).

Because COVI-VAC is more deoptimized and more attenuated than CDX-007, to maximize in vivo safety, we selected it for further study. In COVI-VAC, 1,272 nucleotides of the spike ORF were codon-pair deoptimized for human cells which yielded the 283 silent mutations. In addition, as a second and independent driver of attenuation, the polybasic furin cleavage site in the spike protein of COVI-VAC was removed. Based on published evidence, the elimination of the furin cleavage site is expected to enhance the attenuation profile of COVI-VAC by reducing its infectivity of TMPRSS2-expressing cells in the lower respiratory tract, reducing its capacity for direct cell-to-cell spread by syncytia formation, and increasing its sensitivity to innate immune responses—all features highly desirable in a LAV (22–25).

We examined COVI-VAC growth at 33 and 37 °C in Vero cells (World Health Organization [WHO] 10-87) (Fig. 1*C*). Titers of >1 × 10^7^ plaque forming units (PFU)/mL were reached for WT WA1 grown at both 37 and 33 °C, but titers at that level were reached sooner for cultures grown at 37 °C. In contrast, COVI-VAC grew more slowly at 37 than 33 °C. The robust growth at 33 °C ensures that COVI-VAC can be easily scaled for mass production, while the reduced growth at 37 °C adds another layer of in vivo safety to the potential vaccine.

To investigate the in vivo properties of COVI-VAC, we turned to Syrian golden hamsters (*Mesocricetus auratus*). A recent survey of animal models indicates that these hamsters recapitulate many of the characteristics of human COVID-19 disease and are a relevant small animal model for COVID-19 (26). SARS-CoV-2 replicates efficiently in hamster lungs, causing severe pathological lesions following intranasal infection (27). As has been published, viral antigens are present in nasal mucosa, bronchial epithelium, and areas of lung consolidation on days 2 and 5 after SARS-CoV-2 inoculation, and they are cleared on day 7 (28). Clinical signs include rapid breathing, weight loss, histopathological changes in the lung/airway, intestinal involvement, spleen and lymphoid atrophy, and cytokine activation within 1 wk of virus challenge (29). Infected hamsters can infect other hamsters housed in the same cage, and neutralizing antibodies (Abs) are detected on day 14 postchallenge (29).

**Fig. 2.**
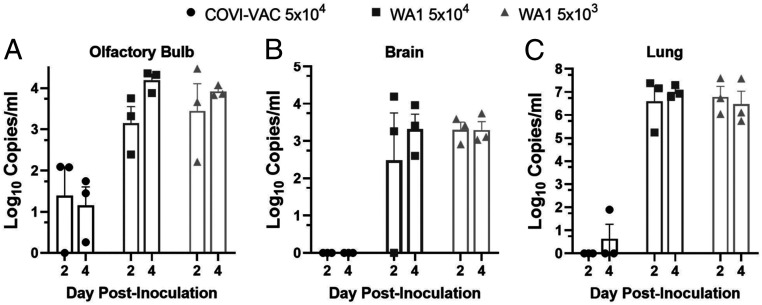
In vivo attenuation of COVI-VAC in hamsters. Hamsters were inoculated with 5 × 10^4^ or 5 × 10^3^ PFU of WT WA1 or 5 × 10^4^ PFU COVI-VAC. Viral RNA was measured by qPCR at days 2 and 4 PI in the (*A*) olfactory bulb, (*B*) brain, and (*C*) lungs. (*n* = 3/group; Bars = SEM).

**Fig. 3.**
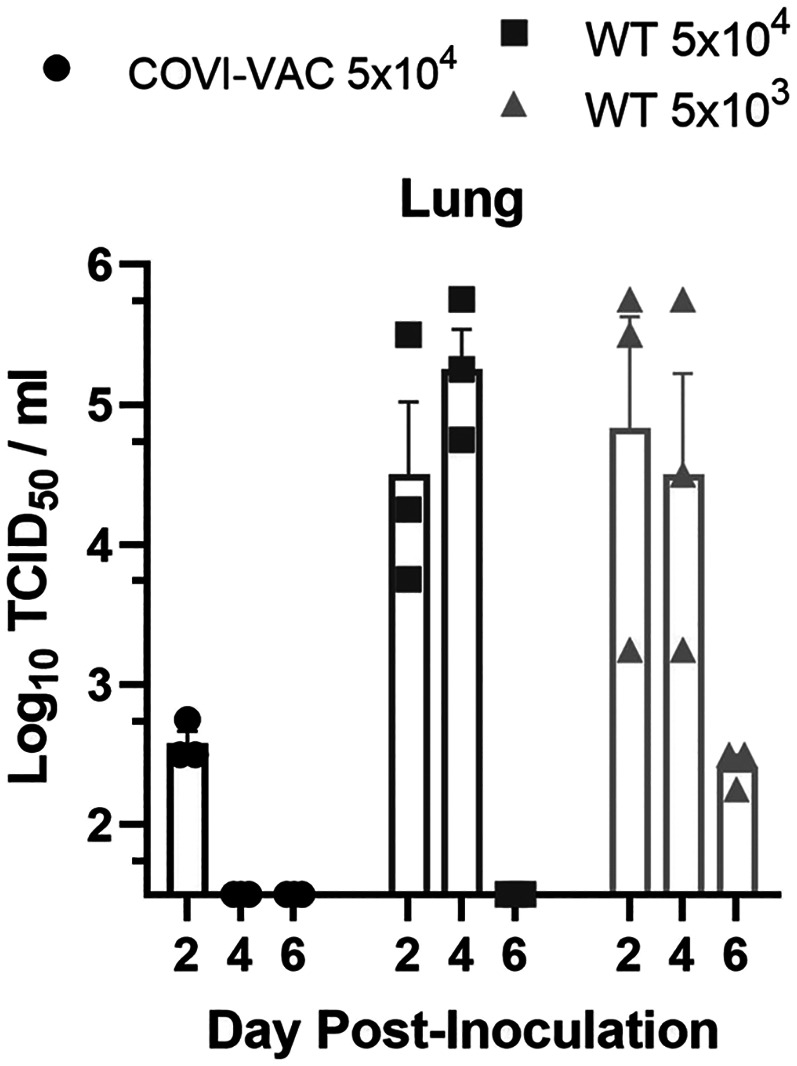
Infectious viral load in COVI-VAC–inoculated hamsters. Hamsters were inoculated with 5 × 10^4^ or 5 × 10^3^ PFU of WT WA1 or 5 × 10^4^ PFU COVI-VAC. Infectious viral load in lung tissue was assessed by TCID_50_ assay and expressed as log_10_ of TCID_50_/mL. Differences between COVI-VAC and WT WA1–treated groups were significant (*n* = 3/group; *P* < 0.01 on days 4 and 6; Bars = SEM).

We evaluated the attenuation, safety, and efficacy of COVI-VAC in 5- to 6-wk-old hamsters under BSL-3. Hamsters were dosed IN on day 0 with 0.05 mL of either 5 × 10^4^ or 5 × 10^3^ PFU/mL WT WA1 or 5 × 10^4^ PFU/mL COVI-VAC. Because COVID-19 disease is associated with pulmonary, olfactory, and neural dysfunction (30–32), we measured viral load in homogenized lungs, olfactory bulbs, and brains. Total viral RNA measured by qPCR was near or below the limit of detection in lung, olfactory bulb, and brain on days 2 and 4 postinoculation (PI) in COVI-VAC–inoculated hamsters. In contrast, viral RNA was detected in WT WA1–infected hamsters in all three tissues at both times (Fig. 2 *A*–*C*). To test if infectious virus was present, we performed tissue culture infective dose 50 (TCID_50_) assays on lung homogenates on days 2, 4, and 6 PI (Fig. 3). By day 4, infectious virus loads were below the limit of detection (<32 TCID_50_/mL) in the lungs of animals inoculated with COVI-VAC, whereas they remained greater than 10^5^ and 10^4^ TCID_50_/mL in animals infected with WT WA1 at 5 × 10^4^ and 5 × 10^3^ PFU, respectively (*n* = 3/group; *P* < 0.01 by factorial ANOVA for independent samples). Thus, COVI-VAC, is highly attenuated relative to WT WA1 in vivo.

**Fig. 4.**
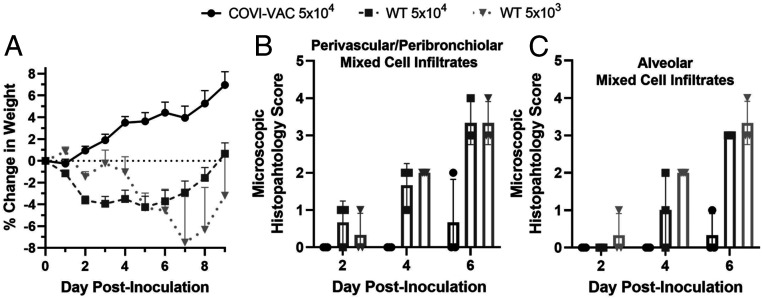
Safety of COVI-VAC in hamsters. Hamsters inoculated with 5 × 10^4^ or 5 × 10^3^ PFU of WT WA1 or 5 × 10^4^ PFU COVI-VAC. (*A*) The weight of hamsters was measured daily for 9 d. Weight changes were significantly different between COVI-VAC– and WT WA1–treated groups (*n* = 10 to 40/group for COVI-VAC and WT WA1 5 × 10^4^; *n* = 3 to 12/group WT WA1 5 × 10^3^; *P* < 0.001; Bars = SEM). (*B* and *C*) Hematoxylin- and eosin-stained lung sections were examined on days 2, 4, and 6 PI and scored on a scale of 0 to 5 for cell infiltration. (*n* = 3/group).

To evaluate the safety of COVI-VAC, the change in weight in hamsters was monitored for 9 d after inoculation. Hamsters inoculated with COVI-VAC experienced weight gain during the period, whereas those inoculated with WT WA1 at either dose experienced weight loss (Fig. 4*A*). Only at day 9 PI did any WT WA1–infected animals return to their starting weight (Fig. 4*A*). A mixed model ANOVA indicated that the changes were significantly different between COVI-VAC– and WT WA1–treated groups (*P* < 0.001).

We also performed histological examinations of the lungs, brain, and kidney. Formalin-fixed paraffin sections were stained with hematoxylin and eosin, and light microscopic evaluation was conducted by a blinded board-certified veterinary pathologist (*n* = 3 per group). Multiple parameters were scored on a 0 to 5 pathology rating scale. No changes were noted in brain and kidney sections of hamsters administered WT WA1 or COVI-VAC. That is, the histopathology scores for all animals in all groups were zero. Consistent with the pathological cellular infiltration found in lungs of humans with COVID-19, however, alveolar and/or perivascular or peribronchiolar mixed cell infiltrates, necrosis of the bronchiolar or bronchial epithelium with neutrophilic infiltration into the lumen, and perivascular edema occasionally accompanied by hyperplasia of the bronchiolar or bronchial epithelium was seen in hamsters infected with WT WA1 (Fig. 4 *B* and *C*). In contrast, lung pathology was limited to minimal alveolar to mild perivascular or peribronchiolar mixed cell infiltrates at day 6 PI in COVI-VAC–inoculated hamsters (Fig. 4 *B* and *C*).

**Fig. 5.**
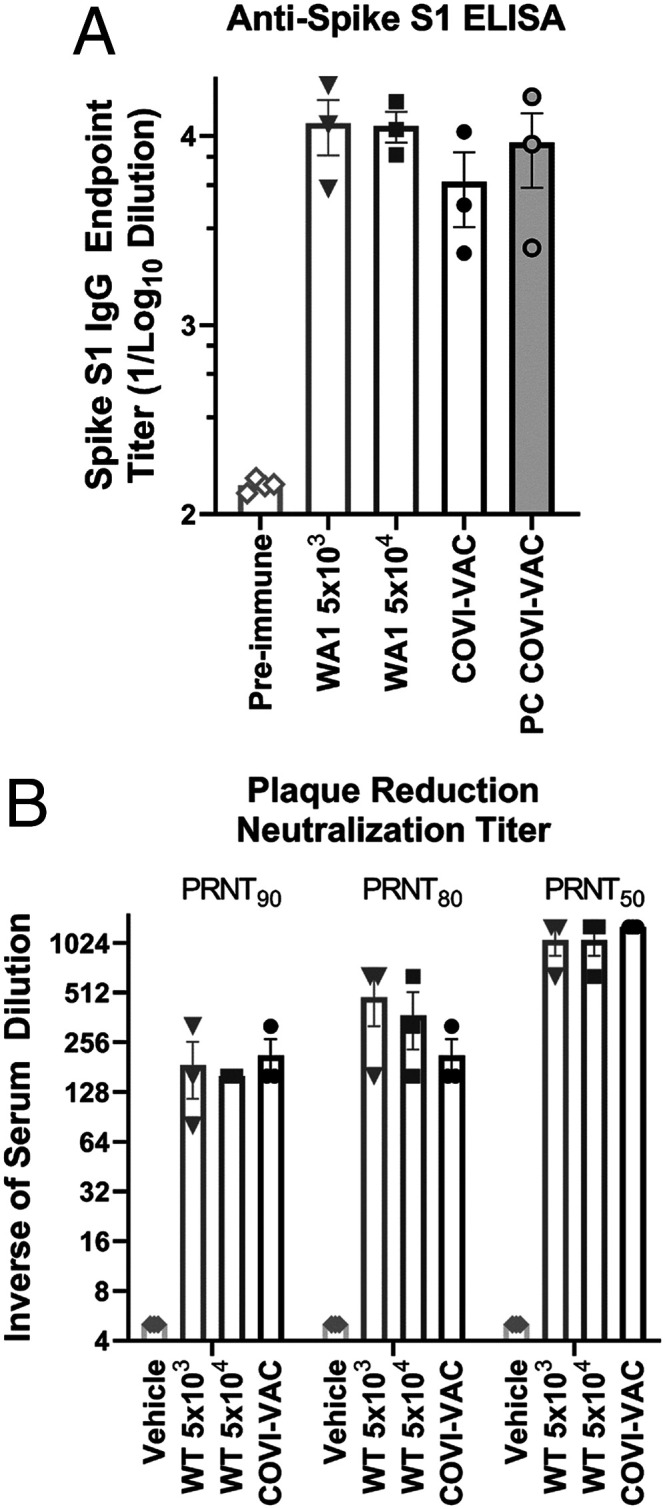
Efficacy in Hamsters. (*A*) A spike-S1 ELISA was performed with preimmune hamster control serum or with serum collected from hamsters on day 16 PI with WT WA1 or 5 × 10^4^ PFU COVI-VAC. Spike S1 IgG was also measured in serum collected on day 18 from COVI-VAC–inoculated hamsters that had been challenged with WA1 on day 16 (PC COVI-VAC). The endpoint IgG titers are shown as the log of the dilution that was 5× above the background. (*n* = 3/group; *P* < 0.01 for all groups versus preimmune; Bars = SEM) (*B*) PRNT against SARS-CoV-2 WA1 were tested in serum of hamsters 16 d after inoculation with 5 × 10^4^ or 5 × 10^3^ PFU of WT WA1 or 5 × 10^4^ PFU COVI-VAC or vehicle. The PRNT is the reciprocal of the last serum dilution that reduced plaque numbers 50, 80, or 90% relative to those in wells containing serum from preimmune hamsters. Titers for vehicle were <eightfold dilution and are plotted as 5 on the graph. (*n* = 3/group; *P* < 0.05 for all groups versus vehicle; Bars = SEM).

To assess efficacy of COVI-VAC as a vaccine, we measured its ability to induce Abs against WT WA1. First, we performed an enzyme-linked immunosorbent assay (ELISA) to determine IgG titers against SARS-CoV-2 spike S1 in control sera and sera of hamsters inoculated with WT WA1 or COVI-VAC. Like WT WA1 infection, COVI-VAC inoculation induced a strong anti-spike S1 Abs response (Fig. 5*A*). We then tested for the presence of neutralizing Abs against WT WA1 virus in serum of inoculated hamsters by plaque reduction neutralization titers (PRNT) on day 16 PI. We calculated 50, 80, and 90% neutralization (inhibition of plaque formation) with serum dilutions up to 1,280-fold. All COVI-VAC–inoculated hamsters produced neutralizing Ab titers at levels similar to those induced by WT WA1 inoculation (Fig. 5*B*).

**Fig. 6.**
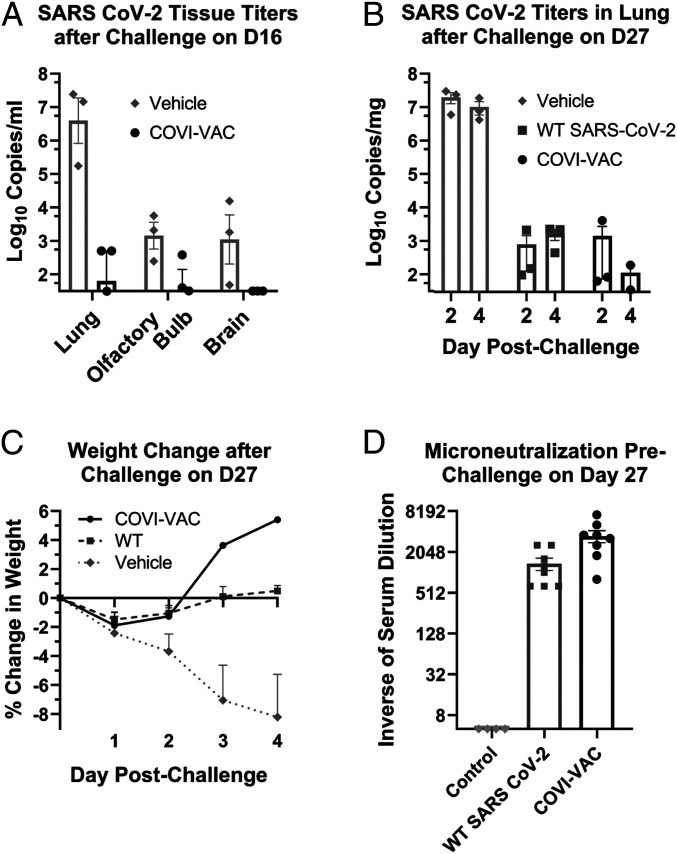
COVI-VAC efficacy. Hamsters were vaccinated with vehicle or 5 × 10^4^ PFU COVI-VAC or *WT* SARS-CoV-2 then challenged IN with 5 × 10^4^ PFU WT SARS-CoV-2 on day 16 (*A*) or 27 (*B*–*D*). The inocula are indicated in the panels. (*A*) Lungs, olfactory bulbs, and brains were harvested 2 d after day 16 challenge. Viral loads were measured by qPCR and expressed as log_10_ of qPCR copies/mL of tissue. The data were plotted on the *x*-axis if below the level of detection (LOD) of 32 copies/mL (*n* = 3/group; *P* < 0.01 for vehicle versus COVI-VAC in lung; Bars = SEM). (*B*) Lungs were harvested 2 or 4 d post challenge at day 27. Viral loads were measured by qPCR and expressed as log_10_ of qPCR copies/mg of tissue. The data were plotted on the *x*-axis if below the LOD of 32 copies/mg. (*n* = 3/group; *P* < 0.01 for vehicle versus both other groups at day 2 and 4; Bars = SEM). (*C*) Weights were recorded on day 27, the day of challenge, and daily for 4 d thereafter. Changes in weights were calculated and plotted as the mean of the percent change in weight for each animal relative to the day of challenge. (*n* = 5 to 6 days 0 to 2, *n* = 3 days 3 to 4, Bars = SEM). (*D*) On day 27, serum from preimmune and immunized hamsters was collected. To assess prechallenge neutralizing antibodies, microneutralization assays were performed. (*n* = 8/group, *P* < 0.05 for COVI-VAC versus both other groups; Bars = SEM).

We measured the efficacy of COVI-VAC in two challenge studies. In the first study, hamsters were vaccinated IN with a single dose of 5 × 10^4^ PFU COVI-VAC and then challenged IN with 5 × 10^4^ PFU WT WA1 on day 16 PI. Lungs were harvested on day 18 (day 2 postchallenge) and viral loads measured by qRT-PCR. Viral loads of the challenge WT WA1 virus were reduced by more than 50,000-fold on average in the lungs of COVI-VAC–vaccinated compared to unvaccinated hamsters, attesting to the efficacy of the vaccine (Fig. 6*A*). Whereas SARS-CoV-2 virus was detected in the brains of challenged naive hamsters, none was detected in COVI-VAC–protected animals (Fig. 6*A*). Levels in the olfactory bulbs were not statistically different in the two groups (Fig. 6*A*). Thus, COVI-VAC conferred protection, and there was no evidence of vaccine-enhanced disease upon challenge. We also found that IgG Abs levels remained high in COVI-VAC–inoculated hamsters at day 2 postchallenge (Fig. 5*A*).

In the second challenge study, hamsters were inoculated as above and challenged with 5 × 10^4^ PFU WT WA1 on day 27 postvaccination. Lungs were harvested on day 29 or 31 (day 2 or 4 postchallenge) and viral loads measured by qRT-PCR. Again, viral loads were significantly reduced by >10^4^-fold at both days (Fig. 6*B*). Efficacy was also evaluated in these animals by measuring the ability of COVI-VAC to protect against SARS-CoV-2–induced weight loss (Fig. 6*C*). Animals inoculated with vehicle exhibited significant weight loss by day 2 postchallenge and continued to lose more weight through at least day 5 postchallenge. In contrast, hamsters inoculated with either WT WA1 or COVI-VAC showed no significant weight loss during the same period. We also assessed neutralizing antibody titers on day 27, just prior to challenge. Microneutralization tests showed that COVI-VAC–immunized animals had >700-fold higher SARS-CoV-2 virus neutralizing antibody titers than at baseline and >2-fold higher than hamsters previously inoculated with WT WA1 virus.

## Discussion

We generated a synthetic highly attenuated live vaccine candidate, COVI-VAC, by recoding portions of the WT SARS-CoV-2 genome according to the *SAVE* algorithm of codon-pair bias deoptimization (7). We suggest that a variety of different mechanisms may collaborate in the attenuation by codon-pair deoptimization: 1) reduced rate of translation, 2) protein misfolding, 3) changes in messenger RNA (mRNA) secondary structures, 4) altered regulatory signals, and 5) increased numbers of 3′-Cp5′-G or 3′-Up5′-A dinucleotides linking two codons. One or all of these processes may contribute to an impairment of genome replication (9). Moreover, many codon-pair–deoptimized viral genomes acquire a temperature sensitive phenotype at 37 °C (Fig. 1*C*) that by itself can be a major contributor to restrict virus proliferation in vivo. Whatever the mechanism, the attenuated COVI-VAC virus presents every viral antigen, providing the potential for a broad immune response and making it likely to retain efficacy even if there is genetic drift in the target strain (3). Also, as with other *SAVE* vaccines, COVI-VAC is expected to be highly resistant to reversion to pathogenicity since hundreds of silent (synonymous) mutations contribute to the phenotype. Our preliminary tests of reversion suggest that the vaccine is stable as assessed by bulk sequencing of late passage virus.

The unique polybasic furin cleavage site present in the WT SARS-CoV-2 spike protein is emerging as a key driver of the lower respiratory tract tropism of the virus and the resulting pathology of COVID-19. While a functional furin cleavage site is not required for efficient SARS-CoV-2 growth in Vero cells, the furin proteolytic processing is critical for efficient infection of TMPRRS2-expressing cells abundant in the lower respiratory tract (24). Further, furin processing of the spike protein may expand the tropism of SARS-CoV-2 to the central nervous system via interaction with neuropilin-1 (33, 34). Finally, the furin cleavage of spike has been suggested to aid SARS-CoV-2 in evading the host innate interferon response (25). Consequently, SARS-CoV-2 viruses without an active furin cleavage site have been shown to be attenuated in small animal models (22, 23) and display reduced capacity of host transmission (35). For these reasons we chose to eliminate the furin cleavage site in the spike protein of COVI-VAC, as a second and independent determinant of its attenuation. While the overall attenuation phenotype of the COVI-VAC strain is likely a combination of both determinants (codon-pair deoptimization and furin cleavage site deletion), we currently cannot say how much either determinant contributes individually.

Like codon-pair deoptimization, the deletion of the furin cleavage is expected to provide a very stable genetic attenuation marker. After 20 passages of COVI-VAC in Vero manufacturing cells, there was no evidence of restoration of the deleted furin cleavage site.

Our hamster studies demonstrate that COVI-VAC is highly attenuated and safe in these animals. It induced lower total viral loads in the lungs and olfactory bulb as well as lower live viral loads in the lung of animals inoculated with COVI-VAC compared to WT WA1. Virus was not detected in brains of COVI-VAC–inoculated hamsters, and unlike WT virus, COVI-VAC inoculation did not induce weight loss or significant lung pathology in infected hamsters.

The hamster studies also suggest that an episode of COVI-VAC infection may effectively protect against illness with SARS-CoV-2. Assessment of Abs titers demonstrate that it is as effective as WT virus in inducing serum IgG and neutralizing Abs. It is protective against WT challenge; inoculation with COVI-VAC leads to lower lung viral titers and complete protection against virus in the brain. Hamsters inoculated with COVI-VAC also do not exhibit the weight loss observed in vehicle-inoculated animals. Moreover, there is no evidence of disease enhancement.

Work remains to be done on characterizing the immune response elicited by COVI-VAC. Studies described here were limited by the capabilities of BSL3 laboratories and the paucity of hamster-specific reagents available early in the pandemic, when this study was conducted. Our current work and the analyses of a Phase 1 clinical trial (ClinicalTrials.gov Identifier: NCT04619628) will shed more light on the profile of the immune response following COVI-VAC vaccination. Regardless of the mechanism of protection, the data presented here demonstrate that, in animals, a single dose of COVI-VAC protects against a WT challenge and promotes the formation of a virus-neutralizing antibody response as potent as that resulting from a WT infection [or possibly more so (see Fig. 6*D*)].

Together, our data suggest that COVI-VAC may be part of a potentially important class of LAVs currently being developed for use in animals and humans (14, 17, 18, 36–38). Like other codon-pair–deoptimized vaccines, it presents all viral antigens with their native amino acid sequence, can be administered IN, is safe and effective in small animal models with a single dose, appears to be resistant to reversion, and can be grown to high titers at a permissive temperature. A Phase 1 clinical trial is currently underway to test its safety and efficacy in humans.

## Materials and Methods

### Virus Production.

LAV candidates against SARS-CoV-2 were generated by recovering viral genomes from WT SARS-CoV-2, strain USA-WA1/2020 (GenBank accession No. MN985325). The reagent was deposited by the Centers for Disease Control and Prevention and obtained through BEI Resources, NIAID, NIH: Genomic RNA from SARS-Related Coronavirus 2, Isolate USA-WA1/2020, NR-52285. To construct two LAV candidates, a portion of the genome-encoding spike gene sequences was replaced by the corresponding codon-pair–deoptimized spike gene sequences (7). COVI-VAC contains 283 and CDX-007 contains 149 silent mutations relative to WT WA1 virus.

#### Transfection of Vero E6 cells by RNA electroporation.

Full-length RNA transcripts of the genomes were in vitro transcribed and electroporated into Vero E6 cells to recover live virus. Vero E6 cells were obtained from the American Type Culture Collection (CRL-1586^TM^) and maintained in high glucose Dulbecco’s modified Eagle medium (DMEM)/10% fetal bovine serum (FBS). Cells were electroporated with 10 µg purified full-length viral RNA transcripts and 5 μg capped WA1-N mRNA using the MaxCyte ATX System (MaxCyte) according to manufacturer’s instructions. Briefly, 3 to 4 × 10^6^ Vero E6 cells were once washed in MaxCyte electroporation buffer, resuspended in 100 µL of the same, mixed gently with the RNA, and transferred to MaxCyte OC-100 processing assemblies. Electroporation was performed using the preprogrammed Vero cell electroporation protocol. After 30-min recovery of the transfected cells at 37 °C/5% CO_2_, cells were resuspended in warm DMEM/10% FBS, seeded at different densities (1/2, 1/3, or 1/6 of the total cells), then incubated at 37 °C/5% CO_2_ for 6 d or until CPE appeared. Infection medium was collected on days 2, 4, and 6, with complete media change at day 2 and day 4 (DMEM/5%FBS). Virus was detectable by plaque assay as early as 2 d post-transfection, with peak virus generation between days 4 to 6.

#### Passaging of stock virus.

Vero E6 cells were cultured in T25 flasks to near confluency. Prior to infection, spent cell culture medium was replaced with 2 mL fresh DMEM without FBS. A total of 100 μL supernatant from infected flasks were added and gently mixed. After a 1-h incubation at 37 °C/5% CO_2_, inoculum was discarded, and fresh complete medium was added. Cells were incubated until CPE was visible.

### Virus Characterization.

#### Plaque titration of SARS-CoV-2 in Vero E6 cells.

Serial 10-fold dilutions were prepared in DMEM/2%FBS. A total of 0.5 mL of each dilution was added to 12 wells of 80% confluent Vero E6 cells. After 1-h incubation at 37 °C, the inoculum was removed, and 2 mL of semisolid overlay of 1× DMEM, 0.3% Gum Tragacanth, 2% FBS, 50 U/mL penicillin, and 50 μg/mL streptomycin added per well. After incubation for 3 or 4 d at 37 °C/5% CO_2_, the overlay was removed, wells were rinsed gently with phosphate-buffered saline (PBS), then fixed and stained with Crystal Violet.

#### Multistep virus growth kinetics.

Vero cells (WHO 10-87) from a “good manufacturing practice” master cell bank established by Charles River Laboratories were grown for 3 d in 12-well plates containing 1 mL DMEM with 5% FBS until they reached near confluency. Prior to infection, spent cell culture medium was replaced with 0.5 mL fresh DMEM containing 1% FBS and 30 PFU of the indicated viruses (multiplicity of infection of 0.0001). After a 1-h incubation at 33 °C or 37 °C/5% CO_2_, inoculum was discarded, cell monolayers were washed once with 1 mL Dulbecco’s PBS, followed by addition of 1 mL DMEM containing 1% FBS. Infected cells were incubated at 33 or 37 °C for 0, 6, 24, 48, or 72 h. At the indicated timepoints, cells and supernatants were collected (one well per time point), frozen once at −80 °C, and thawed. Infectious virus titers in the lysates were determined by plaque assay on Vero E6 at 37 °C.

#### Hamster studies.

Animal studies were performed according to IIT Research Institute and Southern Research’s IACUC-approved protocols.

A total of 36 5- to 6-wk-old male Syrian hamsters (Charles River Laboratories) were anesthetized with ketamine (100 mg/kg) and xylazine (10 mg/kg) via intraperitoneal injection and inoculated IN on day 0 (12 per group) with 0.05 mL of either nominal doses of 5 × 10^4^ PFU/mL or 5 × 10^3^ PFU/mL of WT WA1 SARS-CoV-2 or 5 × 10^4^ PFU/mL COVI-VAC. On day 16, three COVI-VAC–inoculated animals were challenged IN with 5 × 10^4^ PFU/mL WT WA1. Six naïve hamsters inoculated with either 5 × 10^4^ PFU/mL (*n* = 3) or 5 × 10^3^ PFU/mL (*n* = 3) of WT WA1 served as controls. We combined these two control groups for some analyses as titers overlapped at the two inoculation doses.

The 36 hamsters above and an additional 58 (half female/half male) 5- to 6-wk-old Syrian Golden hamsters (Charles River Laboratories) at Southern Research were used to study the effects of COVI-VAC and WT WA1 inoculation on hamster health as assessed by weight loss. The additional 58 hamsters were randomly assigned to one of three groups and inoculated with vehicle or 5 × 10^4^ PFU WT WA1 SARS-CoV-2 or COVI-VAC. All animals were observed twice (day 0 to 8; day 27 to 31) or once (day 9 to 26) daily. In total, 40 5 × 10^4^ PFU COVI-VAC, 40 5 × 10^4^ PFU WT WA1, and 12 5 × 10^3^ PFU WT WA1 were weighed daily for up to 9 d. The N decreased over time for each group as animals were euthanized for other endpoints on various days PI. The minimum N for 5 × 10^4^ PFU COVI-VAC and 5 × 10^4^ PFU WT WA1 was 10 and 3 for 5 × 10^3^ PFU WT WA1.

On day 27, sera were collected from the remaining hamsters. Hamsters were then challenged on day 27. Weights were recorded daily from day 27 (day 0 PC) through day 31 (day 5 PC). By day 28, only five to six hamsters per group remained, due to scheduled tissue sampling throughout the study. For PC weights, *n* = 5 to 6 at day −1 to 3 and *n* = 3 at day 4 to 5 PC.

#### Tissue harvesting.

On days 2, 4, and 6 PI, three hamsters from each group and three hamsters on day 18 from animals challenged on day 16 were euthanized by intravenous injection of Beuthanasia at 150 mg/kg. The left lung was collected for viral load determination. To measure viral load, lung was homogenized in a 10% wt/vol in DMEM with antibiotics using a tissue homogenizer (Omni homogenizer) on day 18 in animals challenged on day 16. We attempted to perform nasal washes but were unsuccessful in obtaining reproducible washes in these small animals.

### Histopathology.

Histopathology was performed by a blinded licensed board-certified veterinary pathologist. Lungs, brains, and kidneys were formalin fixed, dehydrated, embedded in paraffin, and stained with hematoxylin and eosin. Under light microscopy, each tissue was graded on multiple pathological parameters and sections scored as 0 = Normal, 1 = Minimal, 2 = Mild, 3 = Moderate, 4 = Marked, or 5 = Severe. Evaluation of all tissues included assessment of cellular infiltration. At least five sections were examined for each organ and scores averaged.

#### Viral load.

Viral load was measured by qPCR and TCID_50_ in harvested tissue. Tissues were homogenized in a 10% wt/vol in DMEM with antibiotics using a bead mill homogenizer (Omni International). Infectious virus titers were determined by TCID_50_ assay titrating 10-fold serial dilutions of the lung homogenate on Vero E6 cells and are expressed in log_10_ TCID_50_ units/mL. RNA was extracted from 100 µL of brain homogenate using the Quick-RNA Viral Kit (Zymo Research) according to the manufacturer's protocol. qRT-PCR was performed using the iTaq one-step universal probe kit (Bio-Rad) using the following PCR cycling conditions: 40 cycles of 15 s at 95 °C, 15 s at 60 °C, and 20 s at 72 °C.

#### Antibodies–Plaque reduction neutralization titer.

Hamster sera collected at day 16 PI were heat inactivated for 30′ at 56 °C. Two-fold serial dilutions in 50 μL DMEM/1% FBS were performed in 96-well plates, and ∼30 PFU of WA1 in 50 μL DMEM/1% FBS was added to each to a final volume of 100 μL with a total initial serum dilution of 1:10. After incubation at 37 °C/5% CO_2_ for 1 h, the dilutions were added to confluent monolayers of Vero E6 cells (seeded 1 d prior in DMEM/5%FBS in 24-well trays) with 150 μL fresh DMEM/1%FBS. After 1 h virus adsorption at 37 °C/5% CO_2_, 750 μL semisolid overlay was added to the 24-well plates for a final concentration of 1× DMEM, 1.75% FBS, 0.3% Gum Tragacanth, 50 U/mL penicillin, and 50 μg/mL streptomycin in a total volume of 1 mL. Plates were incubated 48 h at 37 °C for plaque formation. Plaques were visualized using 1% crystal violet in 50% methanol/4% formaldehyde. The PRNT 50, 80, and 90 were determined as the reciprocal of the last serum dilution that reduced plaque numbers by 50%, 80%, or 90%, respectively, relative to the plaque numbers in nonneutralized wells containing naïve hamster serum. Sera that failed to neutralize at the lowest dilution (1:10) were assigned a titer of 5, and sera that neutralized at the highest tested serum dilution (1:1,280) were assigned a titer of ≥1,280.

#### Antibodies–IgG ELISA.

The 96-well plates were coated with SARS-CoV-2 (2019-nCoV) spike S1-His (Sino Biological) at 30 ng/well in 50 ng/mL BSA/0.05M carbonate/bicarbonate buffer pH 9.6 overnight at 4 °C. Plates were blocked with 10% goat serum in PBS 2 h at 37 °C, washed 4× with washing buffer (0.1% Tween 20 in PBS), then incubated with a serially diluted serum (1:10 starting dilution and twofold thereafter) in 10% goat serum/0.05% Tween-20 in PBS and incubated 1 h at 37 °C. Plates were washed 4× with washing buffer then incubated with 1:10,000 horseradish peroxidase–conjugated affinity pure goat anti-Syrian hamster IgG (H & L) (Jackson ImmunoResearch Laboratories) for 1 h at 37 °C. Plates were then washed 4× with washing buffer, and *o*-phenylenediamine dihydrochloride (Thermo Scientific) was added to the wells and plates were incubated in the dark at 25 °C for 10 min. Reactions were stopped with 50 μL 2.5M sulfuric acid solution and the absorbance at 490 nm read. Relative IgG levels were reported as the log of the dilution at which the absorbance reached 5× above the no serum control background.

#### Antibodies–Microneutralization.

D27 sera were heat inactivated 45 min at 56 °C, then a series of twofold dilutions in DMEM (Gibco11965)/2% heat-inactivated FBS (Peak Serum) was prepared. Control sera were diluted identically. Diluted sera in a final volume 120 and 60 μL of 2,000 TCID50/mL SARS-CoV-2 (Southern Research) were plated in 96-well trays. Plates were sealed and incubated at 37 °C for 2.5 h. Medium was removed from wells of a 96-well tray that had been seeded with 2 × 10^4^ Vero E6 cells/well the day before, and 100 μL of each serum/SARS- CoV-2 virus mixture added to each of two wells. Plates were covered and incubated 96 h at 37 °C with 5% CO_2_. Plates were removed from incubator and rested at room temperature for 20 to 40 min, then 100 μL of CellTiter-Glo (Promega) prepared according to manufacturer’s instructions was added to all wells, and the plates were gently tapped to mix contents. Plates were incubated 20 min and then read for luminescence.

## Data Availability

COVI-VAC is available for distribution to entities that sign a formal material transfer agreement with Codagenix Inc. COVI-VAC sequence is available on GenBank with accession number MZ404503. The algorithm used to generate the deoptimized viruses was published with the initial provisional US patent application 61/068,666 that can be accessed at “Public PAIR” (https://portal.uspto.gov/pair/PublicPair).
